# Combined Exposure to 33 Trace Elements and Associations With the Risk of Oral Cancer: A Large-Scale Case-Control Study

**DOI:** 10.3389/fnut.2022.913357

**Published:** 2022-07-07

**Authors:** Huiying Wang, Jing Wang, Yujie Cao, Jinfa Chen, Qingrong Deng, Yujia Chen, Yu Qiu, Lisong Lin, Bin Shi, Fengqiong Liu, Baochang He, Fa Chen

**Affiliations:** ^1^Department of Epidemiology and Health Statistics, Fujian Provincial Key Laboratory of Environment Factors and Cancer, School of Public Health, Fujian Medical University, Fuzhou, China; ^2^Key Laboratory of Ministry of Education for Gastrointestinal Cancer, Fujian Medical University, Fuzhou, China; ^3^Laboratory Center, School of Public Health, The Major Subject of Environment and Health of Fujian Key Universities, Fujian Medical University, Fuzhou, China; ^4^Department of Stomatology, The First Affiliated Hospital of Fujian Medical University, Fuzhou, China; ^5^Department of Oral and Maxillofacial Surgery, The First Affiliated Hospital of Fujian Medical University, Fuzhou, China

**Keywords:** oral cancer, trace elements, propensity score matching, mixtures, combined exposure

## Abstract

**Background:**

Trace elements exist widely in the natural environment and mostly enter the human body through drinking water or various types of food, which has raised increasing health concerns. Exposure to a single or a few trace elements has been previously reported to be associated with oral cancer risk, but studies on other elements and combined effects are limited. This study aimed to comprehensively evaluate the independent and joint effects of 33 trace elements on oral cancer risk.

**Methods:**

The concentrations of 33 trace elements from the serum samples of 463 cases and 1,343 controls were measured using inductively coupled plasma mass spectrometry (ICP-MS). Propensity score matching was used to minimize the impact of potential confounders. Conditional logistic regression was utilized to evaluate the association of each element individually with oral cancer risk. Quantile g-computation and Bayesian kernel machine regression (BKMR) models were used to assess the joint effect of the overall element mixture and interactions.

**Results:**

In single-element models, essential elements (Cu, Se, Zn, Sr, and Cr) and non-essential elements (As, Li, Th, Ce, Ti, and Sc) showed significant association with oral cancer risk. In multiple-element models, a quartile increase in overall non-essential elements was observed for a significant inverse association with oral cancer risk (β = −3.36, 95% CI: −4.22 to −2.51). The BKMR analysis revealed a potential beneficial joint effect of essential metals on the risk of oral cancer. Among these, higher levels of serum Zn and V exhibited an adverse effect, while serum Sr, Se, and Cu displayed favorable effects when all other essential elements were fixed at 25th or 50th percentiles. Of note, Se performed complex interactions among essential metals. As for non-essential elements, there were greater effect estimates for serum Th, Li, and Y when all other elements were held at the 75th percentile.

**Conclusion:**

This study provides supportive evidence that the overall mixture effect of essential and non-essential elements might be associated with oral cancer risk, especially for serum Zn, V, Cu, Sr, Se, Th, Li, and Y. Extensive prospective studies and other experiments are warranted to confirm our findings.

## Introduction

Oral cancer, a malignant tumor occurring in various parts of the oral and maxillofacial region, remains a growing public health problem worldwide, especially in the developing countries (1). During the past decade, we and others have conducted a series of epidemiologic studies, demonstrating that smoking, alcohol drinking, betel quid chewing, dietary habits, HPV infection, and poor oral hygiene are important risk factors for the incidence of oral cancer (2–5). However, there are still a certain proportion of oral cancer patients who do not harbor these risk factors, indicating the contribution of other unrevealed factors.

Trace elements are ubiquitously distributed in the natural environment and foods, and have become a global environmental exposure concern gaining increasing attention. Copper, zinc, and selenium are the most representative essential trace elements for the various normal physiological activities and numerous physiological functions (6–8). These essential elements are usually strictly regulated under the normal homeostasis, while the imbalance condition may participate in the occurrence of tumors by increasing cell damage, DNA damage, and oxidative stress (9). Apart from these, other elements (non-essential elements) may also have prominent roles relating to numerous health events. Arsenic, cadmium, beryllium, and other non-essential elements have been reported to exert carcinogenic effects (10–12). These carcinogenic heavy metals are persistent environmental pollutants, as they are not decomposed by microorganisms such as organic pollutants. Therefore, the increasing human extraction and large-scale industrial application of such elements will increase the scale of their migration and transformation in environmental media and ecosystems, and continue to raise the scope, pathways, and doses of human exposure.

Evidence from previous studies has shown that several trace elements may affect similar signaling pathways during tumor development (13, 14). Additional studies have reported that there is a certain antagonistic effect between several trace elements, which will interfere with one’s bioavailability and basic functions by the other (15). However, most of the previous studies focused on one or a few trace elements and associations with oral cancer risk (16–18). To date, limited studies have systematically explored the joint effect of various trace elements on the risk of oral cancer. Considering that we are exposed to various trace elements on a daily basis that are widespread in the natural environment, it is conceivable that the influence of various elements on oral cancer may be a comprehensive mechanism of action. In this study, based on the inductively coupled plasma mass spectrometry (ICP-MS), the most sensitive and promising technique, we detected the serum concentrations of 33 trace elements in a large-sacred sample size, and further explored the independent and joint effects of a variety of serum trace elements on oral cancer risk using two powerful approaches of mixtures analysis [quantile-based g-computation and Bayesian kernel machine regression (BKMR)].

## Materials and Methods

### Study Population

In this hospital-based case-control study, the study participants were recruited from the First Affiliated Hospital of Fujian Medical University (Fujian, China) between November 2010 and August 2019. As previously described (18), 463 patient cases and 1,343 control participants were included; the inclusion criteria for cases were as follows: (1) all cases were those with histologically confirmed primary oral cancer; (2) all cases reside in the Fujian Province at least for 10 years; and (3) all cases aged 20 to 80 years. The exclusion criteria were as follows: (1) patients who have received neoadjuvant chemotherapy or radiotherapy prior to surgery; (2) patients with severe systemic diseases such as liver and kidney dysfunction; and (3) those with long-term dietary supplements. During the same period, control participants were recruited from the health examination center of the same hospital without any history of malignancy. The exclusion criteria were as follows: (1) those who are occupationally exposed to inorganic elements, such as welders and potters; (2) those aged < 20 years or > 80 years; (3) those who did not reside in the Fujian Province; and (4) those who take the long-term dietary supplements.

The studies involving human participants were reviewed and approved by the Institutional Review Board of Fujian Medical University (Approval number: 2011053). The participants provided their written informed consent to participate in this study.

### Data Collection

All participants had signed the informed consent. Face-to-face interview based on standardized questionnaires were used to obtain demographic characteristics, oral health conditions, family history of cancer, frequency of dietary intake, smoking, alcohol drinking, and tea consumption for both case and control by uniform trained investigators. At the end of the survey, the quality of the questionnaires was checked and reviewed by the investigators and failed questionnaires were removed.

### Collection and Detection of Blood Samples

Fasting peripheral blood samples (about 3–5 ml) from oral cancer patients and control participants were collected on the second day of hospitalization before receiving any drugs or examination and the day of the routine health physical examination, respectively. On the day of collection, all samples were centrifuged at 4°C and 3,000 rpm for 10 min to separate the serum and blood cells, and they were stored at −80°C until further analysis.

For measurements of trace elements, first, 200 μl of serum were put into a polytetrafluoroethylene digestion tank, and 1 ml of nitric acid and 4 ml of ultrapure water were added for microwave digestion. Then, excessive acid was removed until the solution was nearly dry. The digested samples were diluted with 5% nitric acid up to 10 ml. Next, all samples are analyzed using inductively coupled plasma mass spectrometry (ICP-MS, NexION 350X; Perkin-Elmer, United States). During detection, if the concentration of an individual element in the sample exceeds the highest concentration point of the standard curve, the concentration of the element should be remeasured after diluting the sample. The concentration of each element below the detection limit is replaced by one-half of the detection limit as suggested in a previous study (19).

### Analytical Quality Control

The human hair powder standard of known concentration (GBW 07601, China) was used as the quality control (QC) sample for continuously monitoring the accuracy of the analytical method. Two reagent blanks and two QC samples were included in each batch, and 12.5% of the samples in each batch were randomly chosen for re-measurement. The relative standard deviation (RSD) of the repeated sample was less than 10%. The limit of quantification (LOQ) was determined based on the 12 replicates of reagent blanks.

### Statistical Analysis

Baseline characteristics between oral cancer patients and control participants were assessed using chi-square analysis. The 1:1 propensity score matching (PSM) within a caliper of 0.02 was carried out to balance the potential confounding between cases and controls. After PSM, group differences were evaluated using standardized mean differences (SMDs), where an SMD value < 0.1 was considered balanced. The serum trace elements were classified into two categories, namely, essential and non-essential. The concentrations of serum essential and non-essential elements were described by the median and 25th (Q25) and 75th (Q75) percentiles among the study population. The concentrations between cases and controls were standardized via ln-transform and compared using Wilcoxon rank sum test. Conditional logistic regression was utilized to estimate the β coefficients and 95% confidence intervals (95% CIs) to evaluate the association between each element individually and the risk of oral cancer.

Next, two-stage mixture analyses were performed. First, as previously described in detail (20), the quantile-based g-computation was used to evaluate the proportion of serum essential and non-essential metals of the overall mixture effect, including positive and negative effect directions. Second, given the possible non-linearity effects of serum elements, BKMR was used to further estimate (a) the exposure-response relationships of one or two elements with the outcome and fixing the remaining metals into different mixture concentrations in essential and non-essential metals, respectively; (b) the cumulative effects on oral cancer compared with mixture median value; (c) the change effect of individual element from the 25th to 75th percentile, while other metal’s concentration at the 25th, 50th, and 75th percentiles. Statistical significance was defined as *p* < 0.05 (two-tailed). All statistical analyses were performed using the R software (version 4.0.5).

## Results

### Subjects’ Demographics Characteristics

Of 463 cases and 1,343 controls enrolled in this study (Table 1), significant differences were observed for gender, age, education level, occupation, residence, tobacco smoking, alcohol drinking, and tea drinking (all *p* < 0.05). Oral cancer patients, compared with their control counterparts, were more likely to have a family history of cancer and below normal BMI. After PSM, 372 oral cancer patients and 372 healthy controls were left for further analysis, and the two groups exhibited comparable demographic characteristics (all *p* > 0.05; SMD < 0.1).

**TABLE 1:** Baseline demographic characteristics of patients with oral cancer.

Variables	Over data			Propensity 1:1 Matching			
	Control (*n* = 1,343) No. of patients (%)	Case (*n* = 463) No. of patients (%)	*P*	Control (*n* = 372) No. of patients (%)	Case (*n* = 372) No. of patients (%)	SMD	*P*
Gender			**<0.001**			0.04	0.553
Male	654 (48.70)	286 (61.77)		209 (56.18)	217 (58.33)		
Female	689 (51.30)	177 (38.23)		163 (43.82)	155 (41.67)		
Age (years)			**<0.001**			0.02	0.825
<60	321 (23.90)	238 (51.40)		165 (44.35)	162 (43.55)		
≥60	1022 (76.10)	225 (48.60)		207 (55.65)	210 (56.45)		
Education level			**<0.001**			0.07	0.904
Primary and below	732 (54.83)	103 (22.44)		100 (27.03)	101 (27.45)		
Middle school	529 (39.63)	293 (63.83)		228 (61.62)	229 (62.23)		
College and above	74 (5.54)	63 (13.73)		42 (11.35)	38 (10.33)		
Occupation			**<0.001**			0.01	0.653
Farmer	613 (45.99)	141 (30.79)		117 (31.79)	129 (34.96)		
Worker	154 (11.55)	70 (15.28)		60 (16.30)	56 (15.18)		
Staff and others	566 (42.46)	247 (53.93)		191 (51.90)	184 (49.86)		
Residence			**<0.001**			0.05	0.502
Urban	1024 (76.25)	257 (55.75)		215 (57.80)	224 (60.22)		
Rural	319 (23.75)	204 (44.25)		157 (42.20)	148 (39.78)		
Marital status			0.995			0.06	0.451
Currently married	1259 (93.75)	434 (93.74)		346 (93.01)	351 (94.35)		
Never married and others	84 (6.25)	29 (6.26)		26 (6.99)	21 (5.65)		
Family history of cancer			**<0.001**			0.01	0.915
No	1221 (91.05)	389 (84.02)		322 (86.56)	321 (86.29)		
Yes	120 (8.95)	74 (15.98)		50 (13.44)	51 (13.71)		
BMI (kg/m^2^)			**<0.001**			0.06	0.710
18.5–23.9	671 (49.96)	298 (64.36)		240 (64.52)	242 (65.05)		
<18.5	66 (4.91)	61 (13.17)		26 (6.99)	31 (8.33)		
≥24.0	606 (45.12)	104 (22.46)		106 (28.49)	99 (26.61)		
Tobacco smoking			**<0.001**			0.03	0.708
No	984 (73.27)	251 (54.21)		226 (60.75)	221 (59.41)		
Yes	359 (26.73)	212 (45.79)		146 (39.25)	151 (40.59)		
Alcohol drinking			**<0.001**			<0.01	1.000
No	1097 (81.68)	291 (62.85)		255 (68.55)	255 (68.55)		
Yes	246 (18.32)	172 (37.15)		117 (31.45)	117 (31.45)		
Tea drinking			**<0.001**			0.03	0.706
No	991 (73.79)	265 (57.24)		227 (61.02)	232 (62.37)		
Yes	352 (26.21)	198 (42.76)		145 (38.98)	140 (37.63)		

*The significance P was bolded.*

### Serum Concentrations of 33 Trace Elements

**FIGURE 1:**
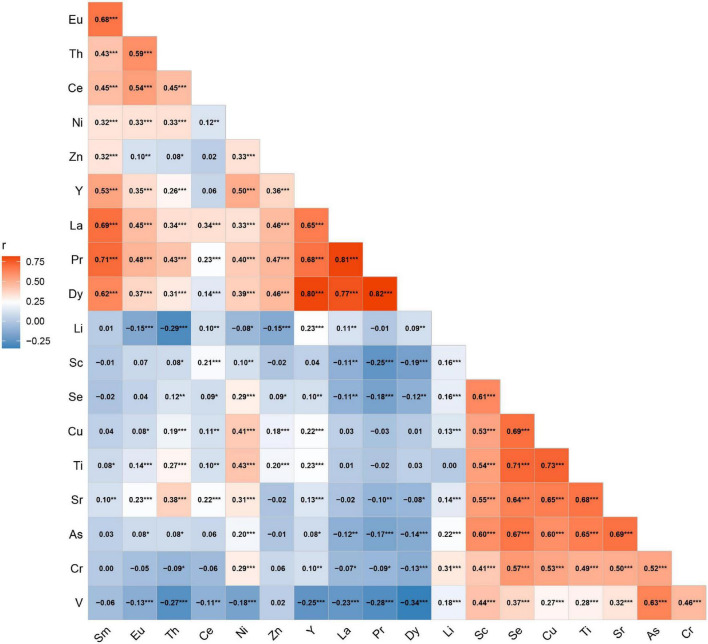
Spearman correlations between serum metals. ***P < 0.001; **P < 0.01; *P < 0.5.

The serum concentrations of 33 trace elements between cases and controls are presented in Table 2. Compared with control participants, oral cancer patients had significantly higher concentrations of Mo, Zn, Cd, Pb, Sb, Pr, Nd, Dy, Sm, La, Ho, Er, Yb, and Lu (all *p* < 0.05). We excluded Cd, Pb, Mo, Co, Nd, Sb, Ho, Er, Lu, Tb, Tm, Yb, Be, and Tl in serum, as their detection rates were less than 50%. Therefore, a total of 19 elements were included in the final analyses. The Spearman correlations between elements are shown in Figure 1. The strongest correlation among essential elements was between Cu and Se (*r* = 0.69), while the strongest correlation among non-essential metals was between Pr and Dy (*r* = 0.82).

**TABLE 2:** Trace metals concentration (μg/L) in sera of all subjects.

Metal	Total (*n* = 744)		Controls (*n* = 372)		Cases (*n* = 372)		LOD	Percentage below LOD (%)	*p*-value*^a^*
	Median	Q_25_–Q_75_	Median	Q_25_–Q_75_	Median	Q_25_–Q_75_			
**Essential elements**									
Cu	1014.04	785.08–1229.66	1116.99	946.87–1272.13	843.09	643.85–1110.08	0.44	0.00	**<0.001**
Mo	0.93	0.48–2.24	0.87	0.44–2.13	1.43	0.83–3.46	0.05	80.38	**0.010**
Mo	0.93	0.48–2.24	0.87	0.44–2.13	1.43	0.83–3.46	0.05	80.38	**0.010**
Se	149.77	114.36–181.61	167.39	145.86–193.65	121.68	84.28–160.00	1.73	0.00	**<0.001**
Zn	1230.40	921.92–1817.81	1029.76	817.38–1295.33	1632.01	1151.85–2744.40	1.60	0.00	**<0.001**
Ni	1.13	0.09–8.04	1.93	0.09–6.48	0.19	0.09–26.84	0.19	45.03	0.624
Sr	51.25	31.99–74.14	69.36	52.98–86.87	34.07	24.27–49.75	0.05	0.00	**<0.001**
Cr	262.22	176.63–341.05	301.61	223.76–370.18	207.83	141.46–287.37	4.06	4.30	**<0.001**
V	34.62	23.67–48.24	34.91	27.16–47.14	33.33	18.37–48.94	0.26	0.00	**<0.001**
Co	1.13	0.06–23.22	–	–	1.13	0.06–23.22	0.03	99.19	–
**Non-essential elements**									
As	28.12	20.75–34.15	31.23	26.07–37.54	23.82	12.75–29.32	0.09	0.00	**<0.001**
Cd	0.15	0.11–0.38	0.12	0.09–0.15	0.56	0.21–0.80	0.05	90.99	**<0.001**
Pb	9.74	4.24–19.94	4.31	2.52–6.80	14.40	7.38–28.07	1.79	62.37	**<0.001**
Li	4.01	0.02–22.08	15.39	0.98–132.91	0.04	0.02–5.99	0.03	36.29	**<0.001**
Sb	2.81	1.06–5.10	2.34	1.16–3.93	3.18	0.90–7.00	0.03	72.45	**0.007**
Th	0.06	0.01–0.19	0.06	0.03–0.35	0.05	0.03 × 10^–1^–0.12	0.01	20.56	**<0.001**
Ce	1.53	0.37–3.15	2.15	0.60–3.86	1.02	0.24–2.74	0.02	4.70	**<0.001**
Pr	0.03	0.00–0.08	0.02	0.02 × 10^–1^–0.03	0.06	0.01–0.13	0.05 × 10^–1^	29.17	**<0.001**
Nd	0.32	0.19–0.57	0.19	0.11–0.25	0.51	0.29–0.77	0.03	58.87	**<0.001**
Ti	329.02	258.19–426.47	351.20	307.54–432.89	278.97	209.97–420.74	0.96	0.00	**<0.001**
Sm	0.04	0.01–0.08	0.03	0.01–0.06	0.04	0.01–0.11	0.02	34.41	**0.002**
Eu	0.01	0.02 × 10^–1^–0.02	0.01	0.02 × 10^–1^–0.02	0.01	0.02 × 10^–1^–0.02	0.03 × 10^–1^	40.46	0.628
La	0.26	0.10–0.57	0.21	0.08–0.37	0.37	0.12–0.82	0.01	6.99	**<0.001**
Y	0.26	0.09–0.45	0.25	0.11–0.39	0.28	0.05–0.56	0.01	11.69	0.357
Sc	6.93	5.31–8.42	7.72	6.52–9.43	5.88	4.27–7.24	0.20	0.00	**<0.001**
Dy	0.02	0.03 × 10^–1^–0.05	0.01	0.03 × 10^–1^–0.03	0.04	0.03 × 10^–1^–0.09	0.01	39.38	**<0.001**
Tb	0.01	0.01–0.02	0.01	0.03 × 10^–1^–0.01	0.01	0.01–0.02	0.02 × 10^–1^	75.81	**0.005**
Ho	0.02	0.01–0.02	0.01	0.03 × 10^–1^–0.01	0.02	0.01–0.02	0.02 × 10^–1^	80.38	**<0.001**
Er	0.04	0.02–0.06	0.02	0.01–0.04	0.04	0.03–0.06	0.01	72.85	**<0.001**
Tm	0.05 × 10^–1^	0.03 × 10^–1^–0.01	–	–	0.05 × 10^–1^	0.03 × 10^–1^–0.01	0.01 × 10^–1^	93.55	–
Yb	0.05	0.03–0.08	0.01	0.01–0.02	0.06	0.04–0.08	0.05 × 10^–1^	73.39	**<0.001**
Lu	0.01	0.06 × 10^–1^–0.01	0.02 × 10^–1^	0.02 × 10^–1^–0.04 × 10^–1^	0.01	0.07 × 10^–1^–0.01	0.01 × 10^–1^	71.37	**<0.001**
Be	–	–	–	–	–	–	0.14	100.00	–
Tl	–	–	–	–	–	–	0.01	100.00	–

*Q25, 25th percentile; Q75, 75thpercentile; LOD, limit of detection; Cu, copper; Mo, molybdenum; Se, selenium; Zn, zinc; Ni, nickel; Sr, strontium; Cr, chromium; V, vanadium; Co, cobalt; As, arsenic; Cd, cadmium; Pb, lead; Li, lithium; Sb, antimony; Th, thorium; Ce, cerium; Pr, praseodymium; Nd, neodymium; Ti, titanium; Sm, samarium; Eu, europium; La, lanthanum; Y, yttrium; Sc, scandium; Dy, dysprosium; Tb, terbium; Ho, holmium; Er, erbium; Tm, thulium; Yb, ytterbium; Lu, lutetium; Be, beryllium; Tl, thallium.*

*^a^P-values were obtained from Wilcoxon rank sum test. The significance P-value was bolded.*

### Conditional Logistic Regression Analysis

**FIGURE 2:**
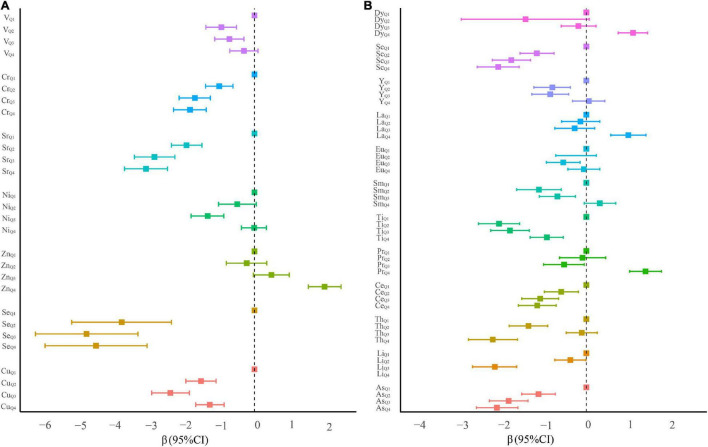
β coefficients and 95% confidence intervals of conditional logistic regression models predicting oral cancer in quartile categories of ln-transformed serum element levels. **(A)** Essential serum metals. **(B)** Non-essential serum metals.

For serum essential elements, high levels of serum Cu, Se, Sr, and Cr, compared with their lowest quartile, were associated with the decreased risk of oral cancer (Figure 2A and Supplementary Table 1). A similar inverse association was also observed for quartile 2 and quartile 3 of serum V. Conversely, compared with participants with the lowest quartile of serum Zn, those in the highest quartile had a significantly elevated risk of oral cancer. Additionally, serum Cu, Se, Zn, Sr, and Cr demonstrated a significant trend by adjusting the potential confounders (all *p*_*trend*_ < 0.05).

For non-essential elements, the highest serum levels of Pr (β = 1.41, 95% CI: 1.03–1.79), La (β = 1.00, 95% CI: 0.58–1.42), and Dy (β = 1.11, 95% CI: 0.76–1.46) were positively associated with a risk of oral cancer (all *p*_*trend*_ < 0.001, Figure 2B and Supplementary Table 1). On the contrary, serum As, Li, Ce, Ti, and Sc at any quartiles compared with the lowest quartile were associated with a significantly reduced risk of oral cancer. Several significantly negative associations of serum Sm, Eu, and Y in quartiles 2 and/or 3 (vs. quartile 1) with the risk of oral cancer were also observed.

### Mixture Analysis

#### Quantile G-Computation Analysis

**FIGURE 3:**
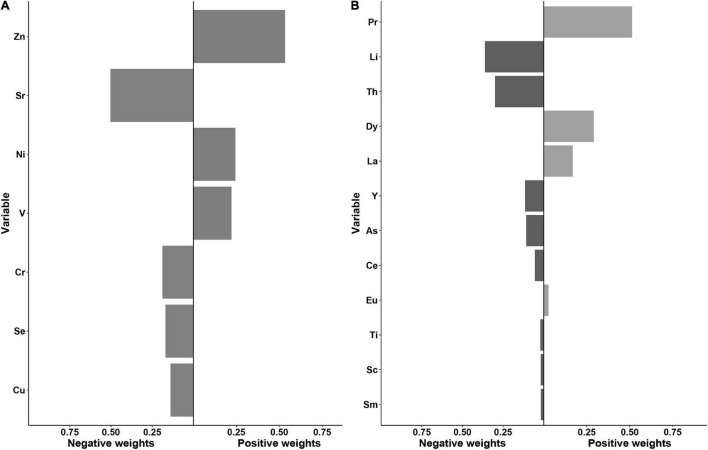
Weights representing the proportion of the positive or negative partial effect for each metal in the quantile g-computation model with **(A)** the essential metals and **(B)** the non-essential metals.

As shown in Figure 3A, using the quantile g-computation mixtures method, serum Sr, Cr, Se, and Cu exhibited negative weights with the same direction in the overall effect of essential elements, whereas positive weights were observed among serum Zn, Ni, and V. Of these essential elements, Zn and Sr were assigned the largest positive and negative weights, respectively. Taken as a whole, increasing all essential metals simultaneously by one quartile did not attain statistical significance (β = 0.10, 95% CI: −0.35 to 0.54, *p* = 0.664, Supplementary Table 2).

With regard to non-essential elements, a significant inverse association with oral cancer risk was observed for all elements combined (the β per quartile increase was −3.36, 95% CI: −4.22 to −2.51, Supplementary Table 2). Serum Li, Th, Y, As, Ce, Ti, Sc, and Sm demonstrated the negative weights, while Pr, Dy, La, and Eu were assigned the positive weights. Among them, Pr and Li showed the largest proportion of the overall effect in the positive and negative directions, respectively (Figure 3B).

#### Bayesian Kernel Machine Regression Analysis

Next, BKMR models with 10,000 iterations after adjusting for the potential confounders further revealed that most of the essential and non-essential elements displayed a non-linear relationship with oral cancer risk (Supplementary Figures 1A,B).

When characterizing the overall effect of the mixture, we observed a decreasing trend in essential metals below their 50th percentile (Supplementary Figure 2A) and in non-essential metals above their 55th percentile (Supplementary Figure 2B), compared with their 50th percentile, respectively.

**FIGURE 4:**
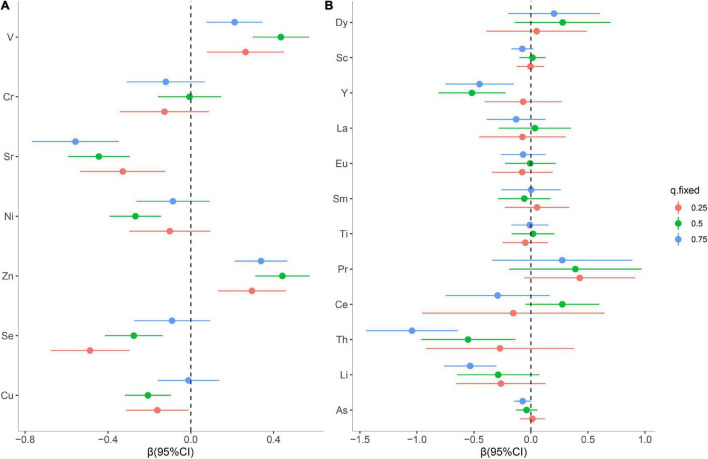
Individual metal risk estimates (95% credible intervals) for each trace metal, while all other metals are fixed at the 25th, 50th, or 75th percentiles for essential metals **(A)** and non-essential metals **(B)**.

Then, the contribution of individual elements to the cumulative effect was further estimated. As shown in Figure 4A, serum Zn and V displayed a significant positive effect, while Sr, Se, and Cu were observed to have a significant inverse effect with oral cancer risk when other essential elements were fixed at 25th and 50th percentiles. Additionally, serum Cu exhibited a significantly inverse association with oral cancer risk when the concentration of Ni was at a low level (Supplementary Figure 3). Of note, a change of Se from its 25th to 75th percentile increases by 0.40 units when the remaining exposures are fixed at their 75th percentile, compared to maintaining them at 25th percentile (Supplementary Figure 5A). As for the non-essential elements, compared with the effect of individual elements, there were greater effect estimates for serum Th, Li, and Y when all other elements were held at the 75th percentile (Figure 4B). Interestingly, in bivariate plots, we observed that Y had a negative relationship at higher Li levels (Supplementary Figure 4), but no statistically significant interaction was found (Supplementary Figure 5B). These findings from the BKMR model were broadly consistent with the results observed in the quantile g-computation analysis.

## Discussion

To the best of our knowledge, this is the first large-scale study to comprehensively evaluate the associations of 33 serum elements and the risk of oral cancer. Using individual element models and mixture analysis based on quantile g-computation and BKMR, we consistently found that individual or mixtures of serum essential metals (Cu, Zn, Se, and Sr) and non-essential metals (Li and Th) were associated with oral cancer risk. We also observed a potentially favorable effect of the essential and non-essential metals at different quantiles, among which, Zn, Sr, Pr, and Li seemingly contribute more weight than other metals.

Accumulating studies have shown that food preferences, cooking methods, element bioavailability across individuals, and environmental background levels of habitat soils would affect individual differences in element accumulation (21–23). Cu, Zn, Sr, and Se are considered essential elements for normal physiological processes, and are involved in the biosynthesis of a number of enzymes (such as ceruloplasmin, zinc-copper superoxide dismutase, and glutathione peroxidase). The deficiency or excessive levels of these metals may affect the enzyme activity (24). In this study, we revealed an inverse association of Cu but a positive association of Zn with oral cancer risk, which was supported by our previous study (16) and is consistent with the nature of the antagonistic effect between Cu and Zn (25). Sr in the human body mainly derives from drinking water and food (26), which could reduce the enamel solubility to prevent the decrease of the hardness of the enamel surface, thus keeping the teeth healthy (27). The observed negative association of serum Sr with oral cancer in this study might be explained by the features of Sr on maintaining good oral hygiene. Additionally, interactive effects and inverse associations for Se were identified in our findings. Se is characterized by antioxidant activity, enhancing immune function and scavenging free radicals (28, 29). Previous studies have indicated that Se could protect against oxidative stress by its immune-modulating and antiproliferative properties, reducing the incidence of head and neck cancer (30, 31). In addition, the effect of Se could be interfered by other elements (15).

We observed a positive association between V exposure and OSCC risk. It has been reported that V would form deposits in the lead pipe for potable water and re-solubilized by phosphate, eventually resulting in health problems (32). In the air, the inhalation of V_2_O_5_ could release proinflammatory factors, causing damage to the respiratory system (32). A recent study also suggested that the concentration of V was significantly associated with the risk of bladder cancer (33). However, other studies pointed out that V^3+^ and V^4+^ have a protective effect from oxidative stress and nephrotoxicity (34, 35). Therefore, it is possible that the relationship between V and the disease may be conditional on the valence of the V ion.

For non-essential metals, we interestingly found that the control group had higher serum As levels, which is consistent with a recent study (36). Inorganic As is a toxic carcinogen for skin, lung, and bladder tumors (37). However, As_2_O_3_ is widely used in clinical cancer treatment, in which reactive oxygen species levels are increased, thus leading to cell death (38). An *in vitro* study by Derakhshan et al. revealed the cytotoxic effect of As_2_O_3_ on oral squamous cell carcinoma (39). Therefore, we speculated that the role of As may vary in different cancer types. Moreover, our findings showed a significant inverse association between Li and oral cancer risk, which was supported by a recent study reporting that LiCl could attenuate oral mucositis and suppress epithelial-mesenchymal transition in tongue mucosa (40). It has also been shown that Li^+^, as an activator of Wnt signaling pathway, could effectively inhibit the proliferation of the ameloblastoma stem cell (41). In addition, we found that Pr had the largest positive weight of the overall effect in non-essential elements. Although there was no straightforward explanation, previous literature provided evidence that Pr might lead to significant DNA genetic damage to Sprague-Dawley rats at a certain concentration (42). As for Th and Y, contrary to our results, previous studies have reported that these elements could cause genetic damage and induce cancer (42–45). However, as radiometal carriers, Th and Y exerted a beneficial action against the carcinoma of head and neck in clinical practice (46, 47). So far, the mechanism between Th, Y, and oral cancer is unclear, which needs further study.

In this study, we used two methods for mixture analysis with multiple metals exposure. Compared with traditional weighted quantile sum, quantile g-computation has significantly faster efficiency and allows that “weights” might go in either direction, directly visualizing each exposure on the positive or negative role of outcome (20). Differing from quantile g-computation, BKMR not only flexibly estimates and plots the exposure-response function that allows non-linear or interactions influences, but also adjusts for potential confounding factors (48). Results from these two powerful methods comprehensively revealed the overall mixture effect or a separate effect of numerous metals on the risk of oral cancer. These findings might provide additional understanding of the underlying pathogenesis of oral cancer.

However, there are some potential limitations in this study. First, we only measure the total metal forms including the organic and inorganic, instead of the specific types. The association between metal ion valence and the risk of oral cancer is warranted to be further explored in the future study. Second, we had performed only a single measurement without duplicate exposure measurements, which may lead to misclassification of exposure. Third, this study was conducted in a single center, and the potential selection bias was inevitable, although the PSM was used to minimize the potential bias. Finally, the case-control study cannot rule out the causal relationship between changes in metals and the occurrence of oral cancer, and further prospective studies are still needed to confirm our findings.

## Conclusion

Our findings suggested that the association between multiple elements exposure and oral cancer risk might vary from essential elements to non-essential elements. Essential trace elements such as Zn, V, Cu, Sr, and Se displayed different degrees of contribution on oral cancer risk and interactive effects existed among them. We also observed the non-linear relationship for Li, Th, and Y when other non-essential metals were at the high levels. A comprehensive evaluation of the individual and joint exposure effects of multiple metals might contribute to unveiling the role of chemical mixtures in the etiology of oral cancer. Much more extensive studies are still required to validate these possible associations.

## Data Availability Statement

The data analyzed in this study is subject to the following licenses/restrictions: information that could compromise research participant privacy or consent. Explicit consent to deposit raw data was not obtained from the participants. Requests to access these datasets should be directed to FC, chenfa@fjmu.edu.cn.

## Ethics Statement

The studies involving human participants were reviewed and approved by the Institutional Review Board of Fujian Medical University (Approval number: 2011053). The patients/participants provided their written informed consent to participate in this study.

## Author Contributions

FC, BH, and LL conceived and designed the study. HW, JW, and YCa analyzed the data and wrote the manuscript. JC, YQ, and BS were responsible for recruitment and interview participants. HW, QD, and YCh performed the investigation. FC and BH obtained funding and material support. FL, FC, and BH revised the manuscript. All authors read and approved the final manuscript.

## Conflict of Interest

The authors declare that the research was conducted in the absence of any commercial or financial relationships that could be construed as a potential conflict of interest.

## Publisher’s Note

All claims expressed in this article are solely those of the authors and do not necessarily represent those of their affiliated organizations, or those of the publisher, the editors and the reviewers. Any product that may be evaluated in this article, or claim that may be made by its manufacturer, is not guaranteed or endorsed by the publisher.
