# Supplementary material for: Combined Exposure to 33 Trace Elements and Associations With the Risk of Oral Cancer: A Large-Scale Case-Control Study
Source: Front Nutr. 2022 Jul 7;9:913357. doi: 10.3389/fnut.2022.913357 (PMC9301066; doi:10.3389/fnut.2022.913357)
Supplement: Supplementary file 1 [file Data_Sheet_1.docx]

**Contents**

**Table S1** Association between the concentrations of serum metals and oral cancer.

**Table S2** Quantile g-computation estimates for the change in oral cancer for one quartile increase in metals.

**Figure S1**. Bayesian Kernel Machine Regression univariate exposure-response functions with the 95% confidence bands for a ln unit increase in essential metals (A) and non-essential metals (B) while all other essential and non-essential metals are held at their median, respectively.

**Figure S2**. Oral cancer risk differences and 95% credible intervals at serum metal mixture percentiles relative to the 50th percentiles as reference of essential elements (left) and non-essential elements(right). Dots represent posterior mean risk difference values, black vertical lines represent 95% credible intervals.

**Figure S3** Bivariate exposure-response functions for every two essential metals on oral cancer.

**Figure S4** Bivariate exposure-response functions for every two non-essential metals on oral cancer.

**Figure S5** Interactive effects for each metal, which is defined by the change in the single metal risk summary when all the other metals are held at the 75th percentile compared to when they are held at the 25th percentile for essential elements (A) and non-essential elements (B). The plots show the difference in effect estimate and the 95% credible intervals.

**Table S1** **Association between the concentrations of serum metals and**

| Metal^a^ | β(95%*CI*) | | | | *P* for trend |
| --- | --- | --- | --- | --- | --- |
|  | Q1 | Q2 | Q3 | Q4 |  |
| Essential elements |  |  |  |  |  |
| Cu | ref | -1.52(-1.95, -1.09) | -2.39(-2.92, -1.85) | -1.27(-1.67, -0.86) | **<0.001** |
| Se | ref | -3.77(-5.19, -2.36) | -4.77(-6.22, -3.31) | -4.50(-5.95, -3.05) | **<0.001** |
| Zn | ref | -0.22(-0.80,0.35) | 0.48(-0.03,0.99) | 1.99(1.53,2.46) | **<0.001** |
| Ni | ref | -0.49(-1.02,0.05) | -1.33(-1.80, -0.87) | -0.01(-0.37,0.34) | 0.992 |
| Sr | ref | -1.93(-2.36, -1.49) | -2.84(-3.41, -2.26) | -3.08(-3.69, -2.47) | **<0.001** |
| Cr | ref | -1.00(-1.39, -0.61) | -1.69(-2.14, -1.25) | -1.83(-2.30, -1.37) | **<0.001** |
| V | ref | -0.94(-1.38, -0.51) | -0.71(-1.13, -0.30) | -0.30(-0.70,0.10) | 0.300 |
| Non-essential elements |  |  |  |  |  |
| As | ref | -1.14(-1.54, -0.74) | -1.85(-2.31, -1.39) | -2.13(-2.62, -1.63) | **<0.001** |
| Li | ref | -0.38(-0.75, -0.01) | -2.18(-2.71, -1.66) | NA | **<0.001** |
| Th | ref | -1.38(-1.84, -0.92) | -0.11(-0.48,0.26) | -2.23(-2.81, -1.64) | **<0.001** |
| Ce | ref | -0.60(-1.00, -0.19) | -1.10(-1.54, -0.66) | -1.17(-1.62, -0.72) | **<0.001** |
| Pr | ref | -0.09(-0.64,0.46) | -0.53(-1.02, -0.05) | 1.41(1.03,1.79) | **<0.001** |
| Ti | ref | -2.08(-2.57, -1.59) | -1.82(-2.28, -1.36) | -0.94(-1.34, -0.55) | **<0.001** |
| Sm | ref | -1.13(-1.66, -0.60) | -0.69(-1.12, -0.26) | 0.32(-0.05,0.70) | 0.290 |
| Eu | ref | -0.24(-0.73,0.24) | -0.55(-0.95, -0.15) | -0.06(-0.44,0.32) | 0.144 |
| La | ref | -0.14(-0.59,0.32) | -0.28(-0.75,0.20) | 1.00(0.58,1.42) | **<0.001** |
| Y | ref | -0.81(-1.25, -0.38) | -0.86(-1.30, -0.42) | 0.06(-0.33,0.44) | 0.203 |
| Sc | ref | -1.18(-1.58, -0.77) | -1.79(-2.24, -1.33) | -2.10(-2.60, -1.60) | **<0.001** |
| Dy | ref | -1.45(-2.98,0.07) | -0.19(-0.60,0.23) | 1.11(0.76,1.46) | **<0.001** |
| ^a^ adjusted for age, gender, education level, residence, marital status, occupation, BMI, family history of cancer, tobacco smoking, and alcohol drinking, tea drinking | | | | | |

**oral cancer**.

| **Table S2 Quantile g-computation estimates for the change in oral cancer for one quartile increase in metals.** | | |  |
| --- | --- | --- | --- |
|  |  |  |  |
| Metal Group | β(95%*CI*) | *P* value^a^ |  |
| Essential metals^b^ | 0.10(-0.35, 0.54) | 0.664 |  |
| Non-essential metals^c^ | -3.36(-4.22, -2.51) | <0.001 |  |
| ^a^: Adjusted for gender, age, education, occupation, residence, marital status, family history of cancer, BMI, tobacco smoking, alcohol drinking, tea drinking ^b^: Includes Cu, Se, Zn, Ni, Sr, Cr, V; ^c^: Includes As, Li, Th, Ce, Pr, Ti, Sm, Eu, La, Y, Sc, Dy; | | |  |

**
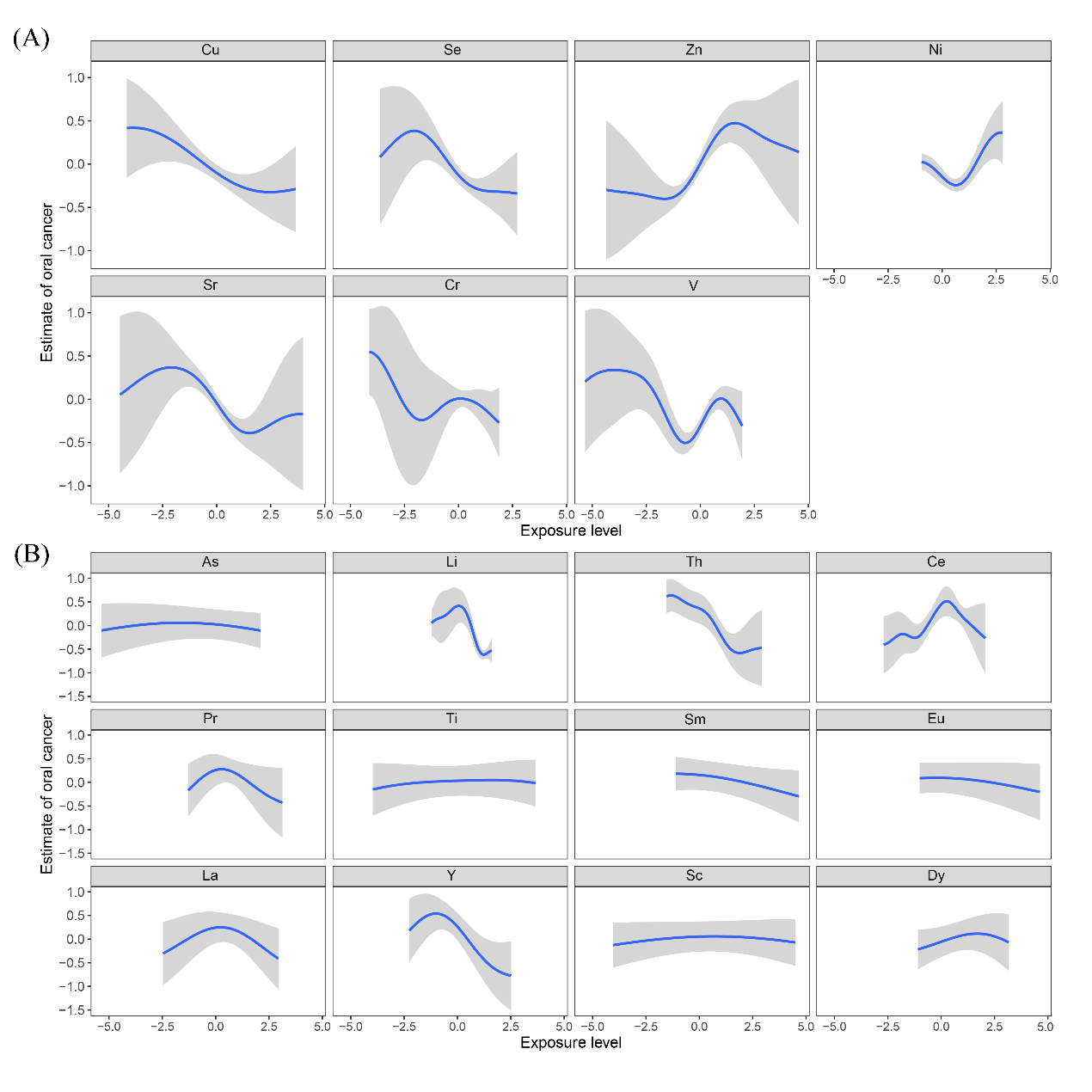
**

**Figure S1.** Bayesian Kernel Machine Regression univariate exposure-response functions with the 95% confidence bands for a ln unit increase in essential metals (A) and non-essential metals (B) while all other essential and non-essential metals are held at their median, respectively.


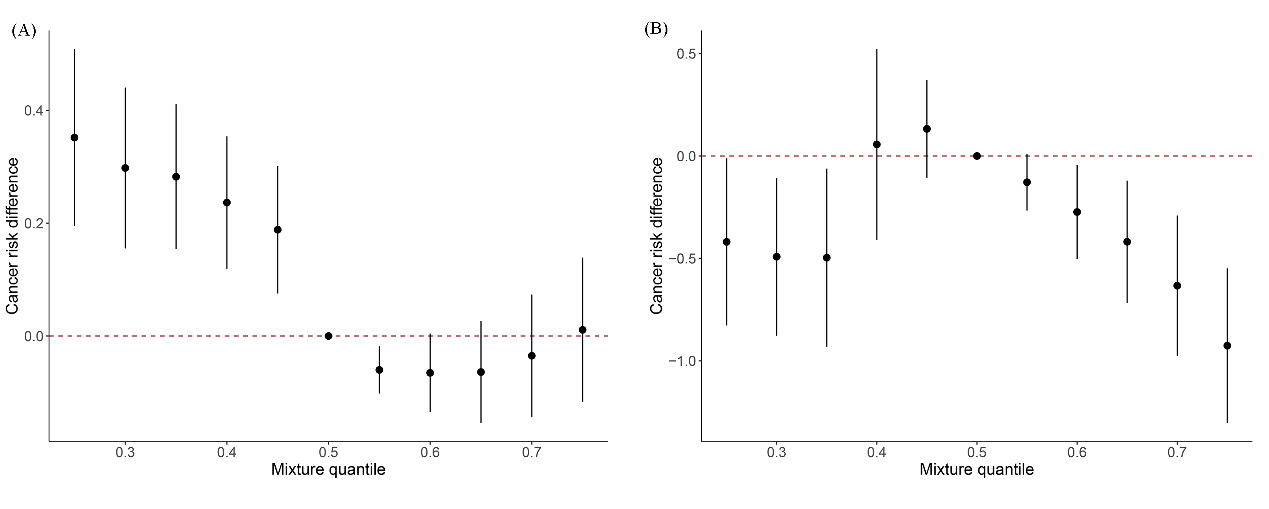


**Figure S2**. Oral cancer risk differences and 95% credible intervals at serum metal mixture percentiles relative to the 50th percentiles as reference of essential elements (left) and non-essential elements(right). Dots represent posterior mean risk difference values, black vertical lines represent 95% credible intervals.

**
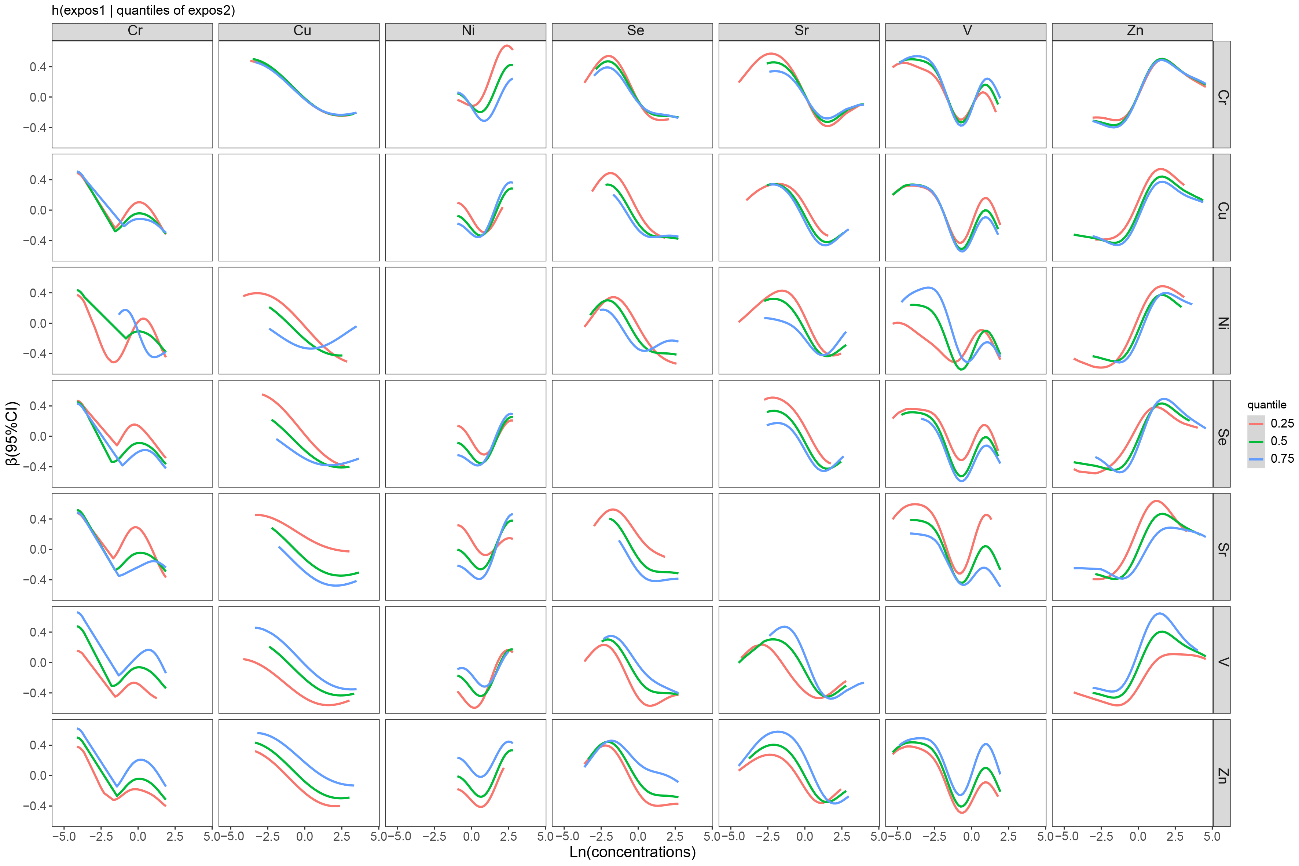
**

**Figure S3** Bivariate exposure-response functions for every two essential metals on oral cancer.


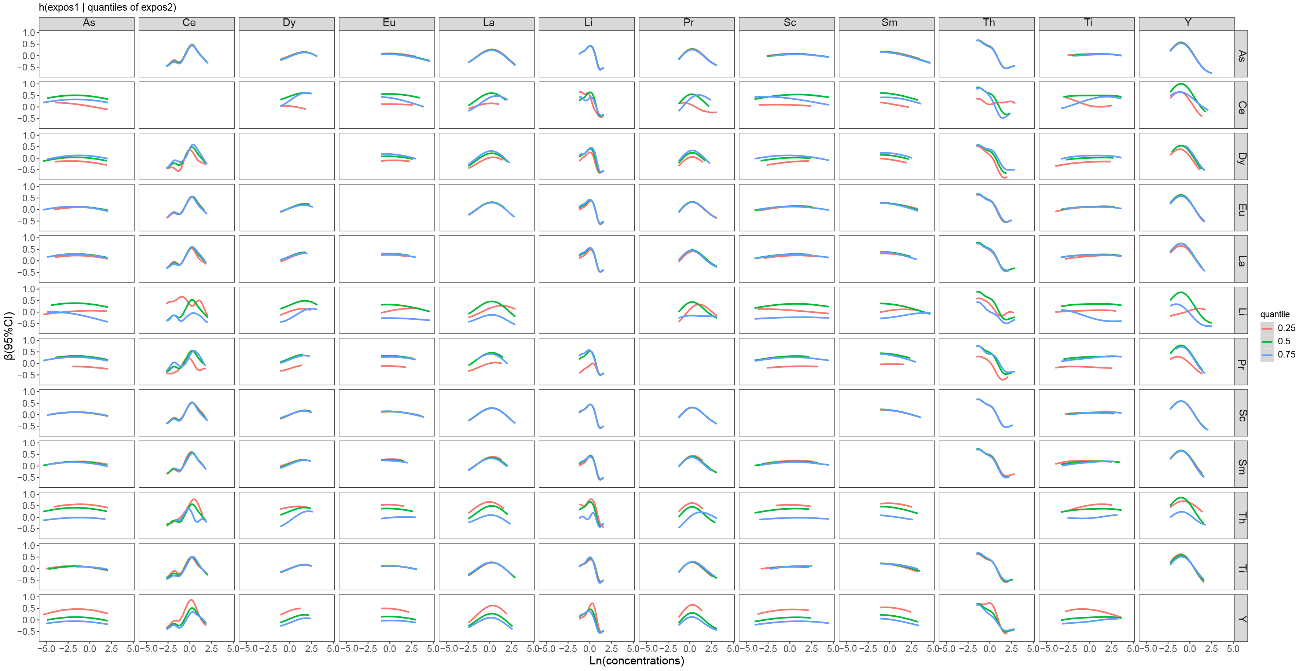


**Figure S4** Bivariate exposure-response functions for every two non-essential metals on oral cancer.

**
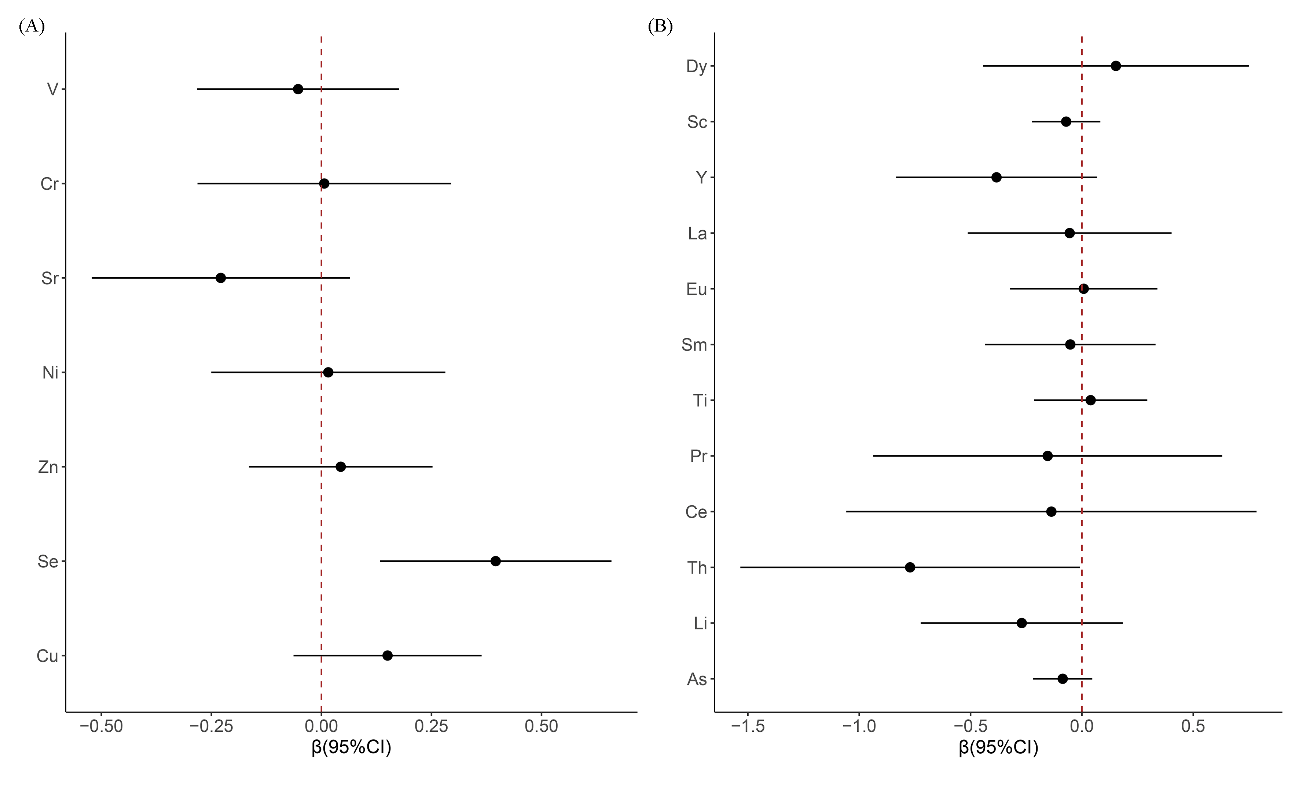
**

**Figure S5** Interactive effects for each metal, which is defined by the change in the single metal risk summary when all the other metals are held at the 75th percentile compared to when they are held at the 25th percentile for essential elements (A) and non-essential elements (B). The plots show the difference in effect estimate and the 95% credible intervals.
